# Neuroprotective efficiency of celecoxib vesicular bilosomes for the management of lipopolysaccharide-induced Alzheimer in mice employing 2^3^ full factorial design

**DOI:** 10.1007/s10787-024-01522-y

**Published:** 2024-07-17

**Authors:** Asmaa Badawy Darwish, Abeer Salama, Mostafa Mohammed Younis

**Affiliations:** 1 Pharmaceutical Technology Department, National Research Centre, 33 El-Buhouth Street, Dokki, Cairo, 12622 Egypt; 2 Pharmacology Department, National Research Centre, 33 El-Buhouth Street, Dokki, Cairo, 12622 Egypt

**Keywords:** Celecoxib (CXB), Bilosomes (BLs), Design-expert, Alzheimer, Sustained release, Histopathological studies

## Abstract

The aim of this study was to develop and evaluate bilosomes loaded with Celecoxib (CXB) for the efficient treatment of Alzheimer. The thin-film hydration approach was utilized in the formulation of CXB bilosomes (CXB-BLs). The study used a 2^3^-factorial design to investigate the impact of several formulation variables. Three separate parameters were investigated: bile salt type (*X*_*1*_), medication amount (*X*_*2*_), and lipid–bile salt ratio (*X*_*3*_). The dependent responses included entrapment efficiency (*Y*_*1*_: EE %), particle size (*Y*_*2*_: PS), and zeta potential (_Y*3*_: ZP). The formulation factors were statistically optimized using the Design-Expert^®^ program. The vesicles demonstrated remarkable CXB encapsulation efficiency, ranging from 94.16 ± 1.91 to 98.38 ± 0.85%. The vesicle sizes ranged from 241.8 ± 6.74 to 352 ± 2.34 nm. The produced formulations have high negative zeta potential values, indicating strong stability. Transmission electron microscopy (TEM) revealed that the optimized vesicles had a spherical form. CXB release from BLs was biphasic, with the release pattern following Higuchi's model. *In vivo* studies confirmed the efficiency of CXB-BLs in management of lipopolysaccharide-induced Alzheimer as CXB-BLs ameliorated cognitive dysfunction, decreased acetylcholinesterase (AChE), and inhibited neuro-inflammation and neuro-degeneration through reducing Toll-like receptor (TLR4), and Interleukin-1β (IL-1β) levels. The findings suggested that the created CXB-BLs could be a potential drug delivery strategy for Alzheimer's treatment.

## Introduction

Alzheimer’s disease (AD) is a degenerative neurological illness that impacts almost 50 million individuals worldwide, creating a severe healthcare, community, and financial challenge (Luo et al. 2013; Tamilselvan and Raghavan 2014) and as populations age and human life expectancy rise, the number of newly diagnosed cases is rising quickly (Cummings et al. 2021). Alzheimer’s disease pathology involves memory loss, spatial disorientation, and a significant deterioration in intellectual skills caused by loss of neurons in higher brain regions (Hassanzadeh et al. 2016; Ouyang et al. 2017).

Although the exact cause of AD is unknown, several mechanisms are believed to be involved, including increased inflammation, ROS accumulation, deposition of amyloid-beta protein, cholinergic insufficiency, tau protein neurofibrillary tangles (NFTs), metal ion dynamic equilibrium problem, and a shortage of neurotropic agents (Ferri et al. 2005; Macauley and Holtzman 2015). Toll-like receptor 4 (TLR4) activation plays a vital role in releasing the inflammation mediators like nuclear factor-kappa β (NF-κβ) and Interleukin-1 β (IL-1β) in Alzheimer’s disease (Elhabak et al. 2023; Salama et al. 2023a, b). Apart from the aforementioned reasons, Alzheimer’s disease may develop and deteriorate due to genetic susceptibility, mitochondrial dysfunction, calcium intoxication, and hormone imbalances (Anand et al. 2014) especially acetylcholinesterase (AChE) elevation (Hegazi et al. 2024).

The selective nature of the blood–brain barrier (BBB) limits the admission of a wide number of central nervous system (CNS) drugs, making a difficulty in treating and preventing Alzheimer’s disease (Sampson et al. 2014; Jiao et al. 2016) given that the BBB is among the human body’s most sophisticated biological obstacles. As a result, molecules with a tiny, positive charge, lipid solubility, and low molecular weight (Mwt) can only pass BBB and enter the brain (Chigumira et al. 2015; Kang et al. 2019). Conventional therapies, such as acetylcholinesterase inhibitors, generally fail due to poor solubility, low bioavailability, and lack of barriers in the brain and bloodstream (Abbas 2021). Therefore, in order to effectively treat AD, medications need to cross the blood–brain barrier (BBB) and reach the brain. The fact that many CNS drugs must be taken at large doses in order to achieve appropriate therapeutic efficacy, which might result in substantial peripheral side effects, is another barrier to the treatment of brain diseases (Karthivashan et al. 2018).

It is known that cyclooxygenase 2 (COX-2) is a crucial enzyme in inflammation and prostaglandin formation which is constitutively produced in neurons (Samad et al. 2001; Tyagi et al. 2020) and increased in AD patients (Hoozemans et al. 2004; Hoozemans and O'Banion 2005). It has been observed that the frontal cortex of AD patients has shown elevated COX-2 levels, especially in neurons that have neurofibrillary tangles (Oka and Takashima 1997). It has also been demonstrated that inflammatory cytokines quickly induce the expression of COX-2 in the cerebral cortex of animal models of AD (Kaufmann et al. 1996). Moreover, it has been demonstrated that COX-2 overexpression correlates with amyloid plaque density and is linked to increased Aβ generation through a prostaglandin E2-mediated mechanism (Qin et al. 2003). These findings suggest that COX-2 plays an essential role in AD neuropathology, and hence COX-2 inhibitors may have a therapeutic effect in AD treatment (Soininen et al. 2007). Studies suggested that the use of nonsteroidal anti-inflammatory medicines (NSAIDs) may prevent or decrease the progression of Alzheimer’s disease (AD) due to their anti-inflammatory characteristics (Kumar et al. 2020).

Celecoxib (CXB), a specific COX-2 inhibitor, is among the most widely used nonsteroidal anti-inflammatory drugs (NSAIDs) for the management of different inflammatory conditions, such as rheumatoid arthritis, osteoarthritis, and ankylosing spondylitis (Arslan et al. 2023). It has been reported that CXB has the ability to reduce neuro-inflammation and thus can prevent cognitive impairment and behavioral abnormalities accompanied with Alzheimer disease (Mhillaj et al. 2020). It is classified as a BSC class II drug due to its lipophilic nature, high permeability, and limited aqueous solubility. CXB has different absorption profiles and a delayed onset of effect lasting 3 to 4 hours after oral dosing. Furthermore, CXB undergoes hepatic first-pass metabolism and is rapidly eliminated from plasma (Cruz et al. 2022). Like other COX-2 inhibitors, CXB has many adverse effects as its long-term use can lead to gastrointestinal irritation and cardiotoxicity. Hence, there is an urgent need for a modified delivery techniques to improve the therapeutic efficiency of CXB with minimizing the incidence of adverse events following its oral administration (Alaaeldin et al. 2021).

Oral medication administration is frequently regarded as the ideal method of drug administration due to its ease of use and patient acceptability. However, there are numerous barriers to this route of administration, including the gastrointestinal tract’s (GIT) acidic environment, enzymatic degradations, the intestine’s changing pH, and mucus secretions, which reduce the medications’ bioavailability and absorption (Stojančević et al. 2014). Factors like water solubility, rate of dissolution, permeability across biological membranes, pre-systemic metabolism, and sensitivity to efflux mechanisms all have an additional impact on the oral bioavailability of medications (Gomez-Orellana 2005). Approximately 60–70% of drug molecules have very low permeability or are not sufficiently soluble in water solutions to be effectively absorbed from the gastrointestinal tract following oral delivery (Gupta et al. 2013). Drug absorption and dissolution rate are significantly impacted by lipophilic medicines’ poor aqueous solubility since maximal absorption requires drug dissolution within the intestinal transit time (Jain et al. 2015). Therefore, it was necessary to create novel drug delivery methods that would maximize a medication's oral absorption and bioavailability while reducing its adverse effects.

The development of nanotechnology-based carriers for brain delivery, such as gold nanoparticles, chitosan, liposomes, micelles, bilosomes, and dendrimers, may hold promise for treatment of various brain diseases. Numerous nanoparticles (NPs) have been investigated for their potential as controlled and targeted drug delivery systems. Excellent stability, drug loading capacity, the ability to integrate both hydrophilic and hydrophobic compounds, and a range of administration options, including oral and inhalation routes of administration, are just a few of the technological advantages of nanoparticles (Gelperina et al. 2005).

Recently, researchers have concentrated on design of new carriers that can mimic the components of biological environment. Bile salt-based nano-vesicles have lately been identified as an important strategy for improving the *in vivo* efficiency of traditional vesicular carrier structures (Verma et al. 2019). Bile salts (BS) are endogenous surfactants with strong solubilization and emulsification properties. Pharmaceutically, they are utilized to increase the solubility of hydrophobic medicines and improve penetration across biological membranes (Elnaggar 2015; El-Nabarawi et al. 2019). Bile salt integration into the lipid bilayer of vesicular carriers has the ability to improve its stability results in self-assembling structures known as “bilosomes” (Zafar et al. 2021). In comparison to other vesicular nanocarriers like liposomes, niosomes, and transferosomes, bilosomes (BLs) exhibit greater chemical and physiological stability and don't require specific storage situations (Abbas et al. 2021). Bile salts' integration into the structure of the vesicular system makes lipid bio-membranes more flexible, enabling them to resist the damaging effects of bile acids in the gastrointestinal tract (GIT). Consequently, the vesicles can shield the medications that are contained therein from the harsh GIT environment. (Conacher et al. 2001). Sodium glycocholate, sodium deoxycholate (SDC), and sodium taurocholate (STC) are examples of bile salts that may serve as penetration enhancing agents. This can improve the movement of lipophilic pharmaceuticals through biological membranes, thereby increasing the oral bioavailability of drugs with limited water solubility and permeation (Aburahma 2016; Elnaggar et al. 2019).

The current study is going to investigate the ability of BLs to improve the efficacy of CXB in Alzheimer’s treatment. The prepared CXB-loaded BLs (CXB-BLs) underwent extensive in vitro characterizations. The goal is to create a formula with a desirable particle size, high drug entrapment efficacy, and optimal drug release. The study attempts to assess the therapeutic virtue of the optimized CXB-BLs in the management of Alzheimer’s disease in a mice model by studying the levels of cognitive dysfunction, acetylcholinesterase (AChE), Toll-like receptor (TLR4), and Interleukin-1β (IL-1β), in addition to the histological examination.

## Materials and methods

### Materials

#### Chemicals

Celecoxib (CXB) was a generous gift from European Egyptian Pharm. Ind. Company, Alexandria, Egypt, whereas phosphatidylcholine (PC), sodium deoxycholate (SDC), and sodium taurocholate (STC) were obtained from Sigma Chemical Co. (St. Louis, MO, USA). Lipopolysaccharide (LPS) was supplied by Sigma-Aldrich (St. Louis, MO, USA). Acetylcholinesterase (AChE), Toll-like receptor 4 (TLR4), and Interleukin-1 beta (IL-1β) were measured utilizing specific ELISA kits (SunLong Biotec Co., LTD, China). All other compounds and solvents utilized in the investigation were of analytical grade.

#### Animals

For the study, male Swiss mice weighing between 20 and 35 g were chosen. They were kept in plastic cages with filter tops, 12 h of light and 12 h of darkness, 50% humidity, and a 28℃ temperature control. Throughout the experiment, mice were fed a regular pellet meal and had unlimited access to water. The investigation was carried out in accordance with the Animal Research Reporting of In Vivo Experiments (ARRIVE) protocol and the National Institute of Health's guidelines for the use and care of laboratory animals (NIH Publication NO. 8023, modified 1978). Additionally, the study was approved by the Medical Research Ethics Committee (MREC), NRC, Cairo, Egypt, with approval number (13,020,254).

### Methods

#### Experimental design

The formulation components of CXB-BLs vesicles were statistically optimized using a 2^3^ complete factorial experimental design. Eight formulas in all were created. Three independent parameters made up the design: *X*_*1*_ (type of BS), *X*_*2*_ (CXB concentration), and *X*_*3*_ (PC: BS ratio). Each element was assessed on two levels. As indicated in Table 1, the chosen dependent responses are Y_1_: entrapment efficiency (EE %), Y_2_: particle size (PS), and Y_3_: zeta potential (ZP). Design-Expert^®^ (Version 8, Stat-Ease Inc., Minneapolis, MN) was used to modify the experimental design in order to optimize and describe the prepared BLs. ANOVA was used to evaluate the importance of the factors under investigation on the chosen responses as well as the interactions among them. A statistically significant *P* value is one that is less than 0.05.

**Table 1:** 2^3^full factorial experimental design factors and responses of CXB-BLs

	Independent factors			Dependent responses		
Formulae	X_1_: BS type	X_2_: PC:BS ratio	X_3_:CXB (w/w %)	Y_1_: EE (%)	Y_2_:PS (nm)	Y_3_: ZP (mV)
CXB-BLs 1	SDC	3:1	0.05	96.24 ± 1.32	350.8 ± 1.35	−16.2 ± 6.72
CXB-BLs 2	STC			95.64 ± 1.54	352 ± 2.34	−27.1 ± 7.70
CXB-BLs 3	SDC		0.1	97.69 ± 1.81	266.4 ± 2.45	−26.6 ± 6.30
CXB-BLs 4	STC			95.89 ± 1.49	278.2 ± 1.89	−28.6 ± 8.15
CXB-BLs 5	SDC	5:1	0.05	96.22 ± 2.66	284.6 ± 3.25	−29.4 ± 5.34
CXB-BLs 6	STC			94.16 ± 1.91	332.8 ± 3.64	−29 ± 6.84
CXB-BLs 7	SDC		0.1	98.38 ± 0.85	241.8 ± 6.74	−34.8 ± 6.74
CXB-BLs 8	STC			98.28 ± 1.14	253.5 ± 6.82	−30.5 ± 7.85

^*^All results are measured as triplicates representing mean ± SD

#### Preparation of CXB-BLs

BLs have been generated using the thin-film hydration approach by adjusting the type of bile salt, CXB concentration, and phospholipids:bile salt ratio (Khalil et al. 2019; Imam et al. 2021; Mohamed et al. 2021; Salama et al. 2022). Accurately determined amounts of PC, bile salts (SDC or STC), and CXB were dissolved in chloroform in a 100 mL round-bottom flask. The rotating evaporator (Büchi rotavapor-M/HB-140, Technik AG, Switzerland) was used to gently evaporate the organic solvent at a temperature of 56–58 °C while operating under reduced pressure. The thin layer that had developed on the moving flask's inner wall after the chloroform had evaporated was hydrated for 45 min using 10 ml of phosphate-buffered saline (PBS), pH 7.4 (Zafar et al. 2021; Younis et al. 2023). Glass spheres were used to increase vesicle output during hydration (Albash et al. 2019). The developed BLs were sonicated for 10 min using a bath sonicator (Ultra Sonicator, Model LC 60/H Elma, Germany) in order to decrease their particle size (PS), then stored at 4 °C for further examinations (El-Nabarawi et al. 2019).

#### In vitro characterization and optimization of CXB-BLs

##### Drug entrapment efficiency percent (EE %) analysis

The free medication was separated from the generated CXB-BLs by cooling centrifugation (Union 32R, Hanil, Korea) at 4 °C and 5200 × g for 30 min. Ten milliliters of PBS were used to wash the pellets after they underwent another centrifugation. The supernatant was filtered using a Millipore 0.22 nm filter (Millipore, USA). The free CXB concentration was measured spectrophotometrically at λmax 252 nm using a Shimadzu UV spectrophotometer (2401/PC, Japan) (Attala and Elsonbaty 2021). To determine the entrapment efficiency (EE %), the quantity of free CXB in the supernatant was deducted from the total amount of CXB using the method shown below:

$$ {\text{EE }}\% \, = \,\left[ {\left( {{\text{Total amount of CXB}} - {\text{Free CXB}}} \right)/{\text{Total amount of CXB}}} \right]\, \times \,{1}00 $$

Particle Size (PS), polydispersity index (PDI), and zeta potential (ZP) analysis

Mean PS, PDI, and ZP of the developed BLs were measured using the Zetasizer (Malvern Instruments Ltd., UK) following adequate samples dilutions (Aziz et al. 2019; Mostafa et al. 2019). ZP findings were assessed using charged vesicles’ electrophoretic mobility. Each analysis was carried out in triplicate (± SD).

#### Selection of the optimized CXB-BLs

Based on the desirability function, optimized CXB-BLs formulation was found using Design-Expert^®^ software (Mohsen et al. 2023). The optimized CXB-BLs formulation was selected to be with the highest EE% (Y_1_), the lowest PS (Y_2_), and the highest ZP value (Y_3_). The option with the greatest degree of desirability was chosen for further examinations.

#### Characterization of the selected formulation

##### Transmission electron microscopy (TEM)

Using TEM (JEOL Co., JEM-2100, Japan), the morphological characteristics of the chosen CXB-BLs formulation were investigated. One drop of the diluted sample was applied to a copper grid coated with carbon to stain the samples, and it was then allowed to dry for fifteen minutes at room temperature. After applying a drop of 1%w/v phosphotungstic acid solution to the grid, it was let to stand for three minutes. After that, the samples were loaded into the microscope and examined at different magnifications for examination of surface characteristics and shape.

##### Fourier-transform infrared (FT-IR) spectroscopy analysis

A FT-IR spectrophotometer (JASCO 6100, Tokyo, Japan) was used to investigate the optimized CXB-BLs in order to find any possible chemical interactions between its components. Sample preparation involved mixing of PC, SDC, CXB, and freeze-dried CXB-BLs with KBr, then compressing the mixture for two minutes at 200 kg/cm^2^ in a hydraulic press. On a blank backdrop made of KBr pellets, each sample KBr pellet was scanned between 4000 and 400 cm^-1^.

##### *In vitro* release study

The dialysis bag method was used to simulate the pH of the gastric and intestinal fluids, respectively, for the release studies of CXB from the optimized Bls formulation and free CXB in 0.1N HCl (pH 1.2) and phosphate-buffered saline (PBS) (pH 6.8) (Kassem et al. 2017; Salama et al. 2023b, a). An amount equal to 2 mg of the CXB-BLs formulation and an aqueous suspension of CXB was placed in the dialysis bags (Dialysis tubing cellulose membrane, Sigma-Aldrich Co., St. Louis, USA; Molecular weight cut-off 12,000 –14,000). To prevent leaking, the bags were sealed on both ends before being placed in 100 ml screw-capped glass containers filled with either 100 ml of 0.1N HCl (pH 1.2) or phosphate-buffered saline (PBS) (pH 6.8) to maintain sink conditions (Mishra et al. 2020). The experiment was carried out in a thermo-stated shaking water bath (Memmert, SV 1422, Germany) at 100 rpm and 37 ± 0.5 °C. To maintain the sink state, samples were taken at predefined intervals (1, 2, 3, 4, 5, 6, 7, 8, and 24 h), and replaced with an equivalent volume of the replacement release medium. The amounts of CXB in the extracted samples were measured and compared to a blank that received the same procedure. The cumulative release percentages were computed by dividing the amount of released CXB by the total amount of CXB in the dialysis bag. Each measurement was repeated three times with different samples.

Kinetic analysis of drug release from the improved CXB-BLs formulation was carried out using various mathematical models, including zero- and first-order kinetic models (Najib and Suleiman 1985), Higuchi’s model (Higuchi 1963), and Peppas model (Basha et al. 2015). The plots of Q versus *t* for the zero order, log Q versus *t*_1/2_ for the first order, Q vs. *t*_1/2_ for the Higuchi model and log Q vs. log t for the Peppas equation were used to determine the *R*^2^ values, which indicate the coefficient of determination where (Q0−Q) is the proportion of CXB left over after time (*t*), and (Q) is the percentage of released CXB at time (*t*). The release exponent “*n*” in Peppas’ model was computed to imply the drug release mechanism.

#### *In vivo* Study

##### Experimental design

Swiss male mice were used in the biological assessment of the chosen formulation’s ability to treat Alzheimer’s disease. Forty animals were divided into five groups randomly (each group contains 8 mice). Group I served as a negative control group. Animals in Group 2 received intraperitoneal injections of 250 µg/kg of LPS an acted as a positive control group (Kim et al. 2017). The mice in groups 3, 4, and 5 were given oral doses of 10 mg/kg of drug-free BLs, free CXB, and CXB-BLs, respectively, for seven days concurrent with LPS administration (Mishra et al. 2020).

##### Estimation of the behavioral activity using Y maze

The behavioral activity experiment was conducted following the study performed by Hidaka et al. (Hidaka et al. 2011) using the Y maze apparatus. There are three arms in the Y maze, and they are all labeled A, B, or C. The maze can be traversed by the animal during the first eight-minute training phase, then it is given eight minutes to move during the second phase, which takes place 24 h later, and its movements are recorded. The number of alternations in overlapping triplet sets (e.g., ABCBACA = 3) is the number of consecutive entries into three separate arms. The term “total arm entries” refers only to the total number of arms inputted (for example, ABCBACA = 7). The following formula was used to determine the percentage alternation (Miwa et al. 2011):

$$ {\text{The percentage alternation }} = \frac{{\text{Number of alternations}}}{{\left( {{\text{Total arm Entries }} - { }2} \right)}} \times {1}00 $$

##### Tissue biochemical analysis

Mice were beheaded and sacrificed. Each mouse brain was dissected right away, and any extra blood was removed with phosphate-buffered saline (PBS). The weighed components were homogenized in PBS using an MPW-120 homogenizer (Med Instruments, Poland) to produce a 20% homogenate, which was then kept cold for the entire night. The homogenates were placed in a cooling centrifuge (Sigma and Laborzentrifugen, 2k15, Germany) and centrifuged for 5 min at 5000 xg (Salama et al. 2016). The supernatant was removed immediately and assayed for brain contents of AChE, TLR4, and IL-1β using an ELISA kit (SunLong Biotec Co., LTD, China) (Salama et al. 2023b, a).

##### Histopathological examination

Mice brains from different groups were autopsied, and the samples were kept for a full day in 10% formaldehyde solution. The subjects were dehydrated using methyl, ethyl, and absolute ethyl alcohol dilutions in order after being cleaned with tap water. Specimens were cleaned in xylene and then placed in a hot air oven at 56 degrees for an entire day. Using a revolving LEITZ microtome, paraffin beeswax tissue blocks were produced for sectioning at a thickness of 4 microns. To be examined using a light electric microscope, the acquired tissue sections were placed on glass slides, deparaffinized, and stained with hematoxylin and eosin (Bancroft et al. 2013).

#### Statistical analysis

Standard deviations (SD) along with means are presented for every data set. The different groups were compared using one-way analysis of variance (ANOVA), and for multiple comparisons, Tukey’s multiple comparisons test was employed. The statistical tests were conducted with the assistance of Graph Pad Prism version 5 (Inc., USA). A difference was regarded as significant when *P* ≤ 0.05.

## Results and discussion

### Preparation of CXB-BLs

In the present research, CXB-BLs were generated using the thin-film hydration procedure. Table 1 shows the composition of the eight formulations, as well as the outcomes of the responses under consideration. BLs are significant delivery vehicles that can be used as an alternative carrier for oral delivery of different types of medications (Elkomy et al. 2022a, b). They are different from liposomes and niosomes as they have superior characteristics, such as low drug leakage, high loading capacity, and delivery through the gastrointestinal tract (GIT) (Elkomy, Alruwaili et al. 2022). Several bile salts have been utilized in the manufacture of BLs. They can serve as permeation enhancers, allowing BLs to cross biological barriers (Waglewska et al. 2020). They can help prevent BLs breakdown in the GI tract, increasing penetration and making oral delivery more effective. Additionally, the BLs’ colloidal stability is increased by the inclusion of certain bile salts (BS), like sodium deoxycholate (SDC), which enables them to resist the disturbing adverse reactions caused by physiological acids in the GIT (Arzani et al. 2015). It has been observed that bile salts increase intestinal epithelial cells’ uptake of vesicles while preventing enzymatic activity at the site of absorption (Aburahma 2016). SDC and STC were employed as bile salts in the manufacture of BLs formulations in the current study.

### Analysis of the factorial design

A software entitled Design-Expert^®^ was used to customize a 2^3^ factorial design. As shown in Table 1, each of the three independent variables was examined twice. ANOVA was used to assess the independent components’ significance, size, and interactions with the proposed responses. *P* values below 0.05 indicate that the model terms are significant. The correlation coefficient (*R*^2^) was used to assess the model's quality of fit.

### Characterization of the developed CXB-BLs

#### Determination of entrapment efficiency percent (EE %)

The EE% findings for all the generated formulations are shown in Table 1. All formulations had high EE% values, ranging from 94.16 ± 1.91 to 98.38 ± 0.85%. Table 1 shows that BLs prepared with SDC had greater EE% values than BLs prepared with STC at the same amount of CXB and PC:BS ratio. This could be owing to a difference in the fluidity of the bilayers, which influences the leaking of the entrapped medication (Elnaggar et al. 2019). Increasing the molar ratio of PC:BS from 3:1 to 5:1 increased the lipophilicity of the developed BLs, resulting in an increase in CXB entrapment (Abdelbary and Aburahma 2015). It has previously been observed that increasing the concentration of BS induces micelles formation throughout the dispersion medium, resulting in better drug solubility and lowering the EE% (Mahmood et al. 2014; Niu et al. 2014). Furthermore, high concentrations of BS have a fluidizing impact on the lipid bilayers of vesicles, causing the encapsulated medication to be released (Ahmed et al. 2020). While the obtained data indicated that with respect to the drug amount factor, the EE% increase as the drug concentration increased from 0.05 up to 0.1%, which might be due to the lipophilic property of CXB which enables it to distribute in the lipid bilayers and get entrapped in the vesicles (Yusuf et al. 2014).

##### Statistical examination of the formulations variables impact on EE% (*Y*_*1*_)

**Fig.1:**
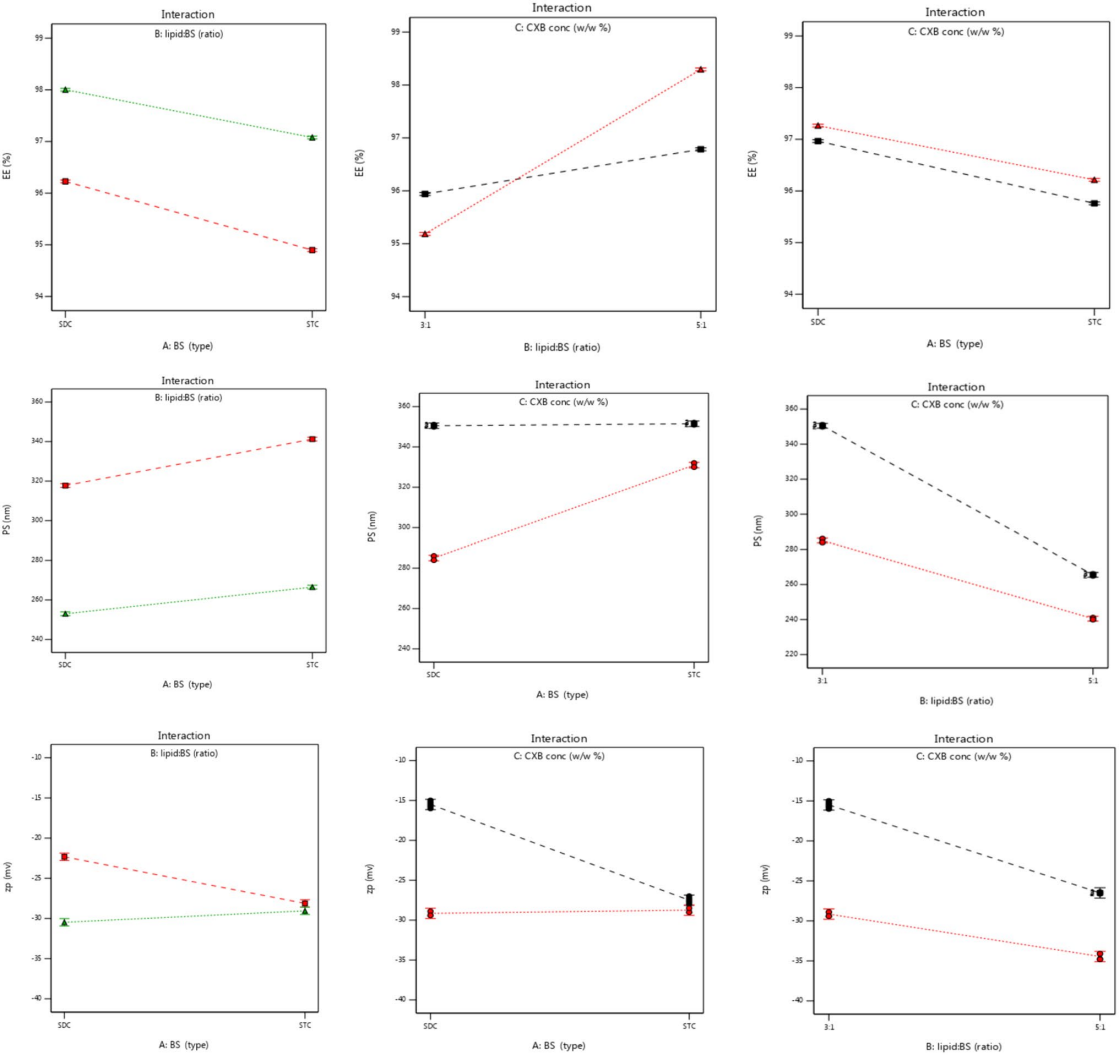
Graphical illustration of the factors interactions on responses; Y1 **a**, Y2 **b** and Y3 **c**.

Analysis of the data in Table 2 by ANOVA shows that EE% was positively and significantly impacted by factors A (BS Type), B (PC:BS ratio), and C (CXB concentration). In Fig. 1, the consequences of each component are illustrated graphically. The correlation coefficient (*R*^2^) = 0.9997, which indicates a well-fitting model, and the model *F* value of 3795.27 proposes the relevance of the model. The effects of all three single components (*X*_*1*_*, X*_*2*_*, and X*_*3*_) are statistically significant (*p* < 0.05) with p values of less than 0.0001, according to the findings of the ANOVA test. Furthermore, Y1 was significantly affected (*p* < 0.05) by the interaction terms (*X*_*1*_*.X*_*2*_*), (X*_*1*_*.X*_*3*_*), and (X*_*2*_*.X*_*3*_). The interaction of the factors on the dependent responses is graphically represented in Fig. 1.

**Table 2:** ANOVA analysis of the dependent variables

Y	SS	*df*	MS	*F* value	*P* value	*R*^2^	Adj. *R*^2^	Pred. *R*^2^
EE %	29.22	7	4.17	3795.27	< 0.0001	0.9997	0.9994	0.9988
PS	26,840.75	7	3834.39	2788.65	< 0.0001	0.9996	0.9992	0.9984
ZP	408.39	7	58.34	185.58	< 0.0001	0.9993	0.9885	0.9755

*SS* sum of square, *df* Degree of freedom, *MS* mean sum of square, *R*^*2*^ determination coefficient, Adj. *R*^*2*^ adjusted determination coefficient, Pred. *R*^*2*^ predicted determination coefficient

#### Particle size (PS), polydispersity index (PDI) of the developed CXB-BLs

The CXB-BLs formulations ranged in size from 241.8 ± 6.74 to 352 ± 2.34 nm (Table 1), indicating they were all nanosized. As shown in Table 1, PS values were lower in BLs prepared with SDC compared to BLs prepared with STC at the same dose of drug and PC. BS ratio. The variations in PS could be related to variations in the structure of the utilized BS (Abdelbary et al. 2016). It has been reported that SDC has molecular weight lower than the molecular weight of STC. Thus, larger particles were prepared with STC utilization (Aburahma 2016). Furthermore, increasing the molecular weight increases the viscosity of the prepared BLs, which may result in aggregation and amplification of the PS (Yang et al. 2013). The ratio of PC:BS is a critical factor that can affect the PS and PDI of formulations (Ahmad et al. 2017). According to the data, regardless of the type of BS utilized, the PS is significantly less when the ratio between PC:BS is 5:1 as opposed to 3:1. This could be due to the vesicles’ superior stability at the former ratio. Previous studies indicated that the use of PC:BS ratio of 5:1 can produce BLs of significantly lower sizes (Chen et al. 2009). In relation to the drug amount factor, the data obtained indicated that the PS decreased as the drug concentration increased from 0.05 to 0.1%. This might potentially be attributed to the hydrophobic character of CXB, which enables it to be trapped between the bilayers of BLs rather than in the hydrophilic core. Additionally, the findings verified modest polydispersity index (PDI) values, which varied from 0.232 ± 0.26 to 0.377 ± 0.32, indicating a confined and uniform range of vesicle sizes.

##### Statistical examination of the formulations variables impact on PS (*Y*_*2*_)

Good collaboration was verified by the experimental PS examinations that were adjusted to fit the conventional least square model, as demonstrated in Table 2 with *p* < 0.0001 and *R*^2^ = 0.9996. According to the findings of the ANOVA test, all three different factors (*X*_*1*_*, X*_*2*_*, and X*_*3*_) have statistically significant impacts (*p* < 0.05). Figure 1 depicts the strong impact of interactions *X*_*1*_*.X*_*2*_*, X*_*2*_*.X*_*3*_*, and X*_*1*_*.X*_*3*_ on the PS (*p* < 0.05).

##### Zeta potential (ZP) of the developed CXB-BLs

Table 1 shows that all CXB-BLs preparations had negative charges ranging from −16.2 ± 6.72 to −34.8 ± 6.74 mV, indicating stable dispersions. The formulations’ negativity revealed that BS were deposited within the phospholipid bilayer of the generated BLs, giving the formulation a negative surface charge (Ahmad et al. 2017; El-Nabarawi et al. 2019). The significantly negative zeta potential could be attributed to the phosphatidic acid and free fatty acids present in the phospholipid (PC), together with the negativity provided by the bile salt impeded in the phospholipid bilayer. This result was similar to Mazer’s theory, which postulated that BS molecules were not just adsorbed on the vesicular surface but were trapped in the PC lipid bilayer (Hu et al. 2013).

##### Statistical examination of the formulation variables impact on ZP (*Y*_*3*_)

The study found that all three parameters and their interactions had a significant impact on the ZP (*p* < 0.05). The least squares model has a strong correlation with the experimental data, with *R*^2^ = 0.9993 and modified *R*^2^ = 0.9885, with a significance level of *p* < 0.05. The ANOVA test revealed statistically significant impacts for all components (*X*_*1*_*, X*_*2*_*, and X*_*3*_) (*p* < 0.05). The interactions *X*_*1*_*.X*_*2*_*, X*_*2*_*.X*_*3*_*, and X*_*1*_*.X*_*3*_ have a substantial influence on ZP (*p* < 0.05) (see Fig. 1).

## Selection of the optimized formulation

**Fig.2:**
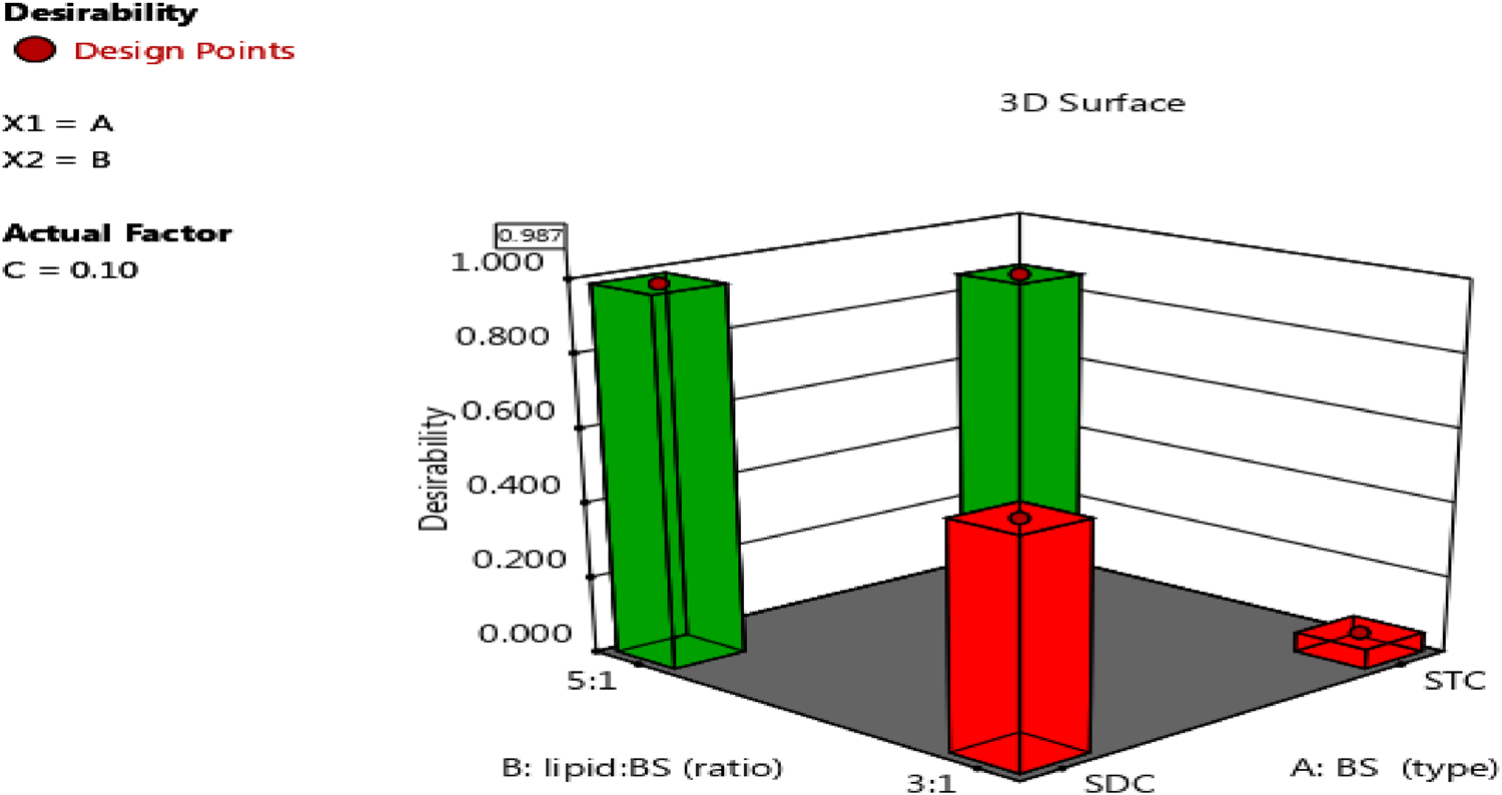
Graphical illustration of the desirability.

Numerical and graphical analyses of the factors influencing the selected responses were used, which aid in the development of suitable BLs formulations under a given set of constraints (Khalil et al. 2019). As a result, optimal formulations should have a higher EE% and a greater magnitude of ZP while reducing PS. Design-Expert^®^ software was used to optimize CXB-BLs for the following responses: Y_1_ ≥ 98%, Y_2_ ≤ 250 nm, and Y_3_ ≥ −30 mV. Figure 2 depicted a graphical representation of the desirability. Finally, *CXB-BLs 7* (0.1% CXB, 5:1 PC:SDC) was identified as the selected BLs vesicles, with a desirability of 0.987, and is going to be investigated in further characterization.

## Characterization of the optimized CXB-BLs

### Transmission electron microscopy (TEM)

**Fig.3:**
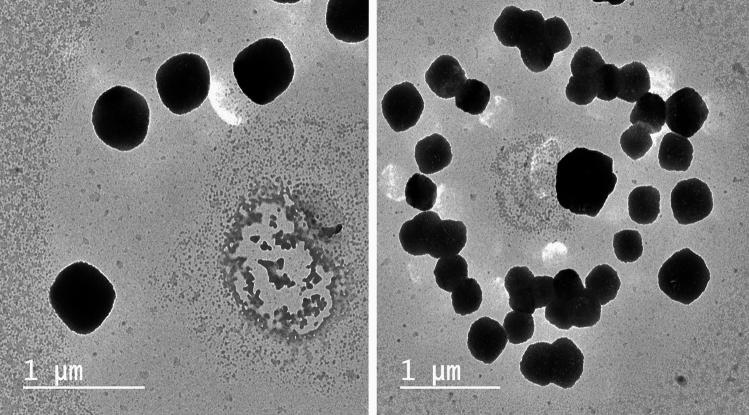
TEM of the optimized CXB-BLs formulation

Figure 3 depicts the shape of selected BLs vesicles CXB-BLs 7. The micrographs revealed the structure of a vesicle comparable to liposomes, with a nearly flawless sphere-like shape, a smooth surface, a generally uniform size, and a well-distributed nature when scattered in an aqueous environment (Salama et al. 2022). The vesicles were found in dispersed and aggregated groups. The electron micrographs showed the outline and core of the vesicles, revealing the presence of impenetrable vesicular structure.

### Fourier-transform infrared (FT-IR) spectroscopy analysis

**Fig.4:**
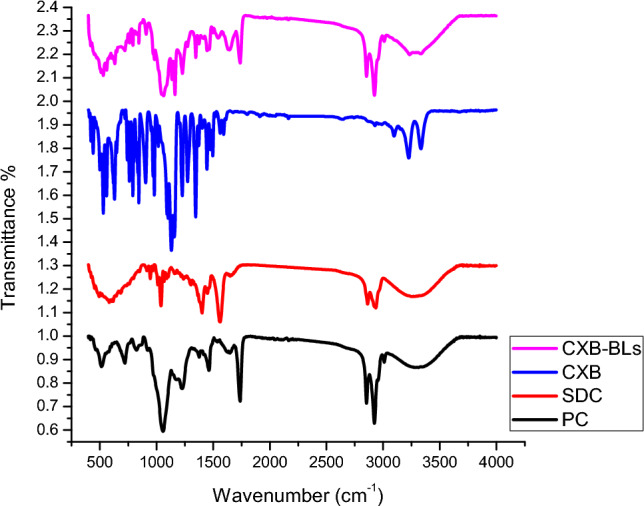
FTIR thermo grams of CXB-BLs formulation and individual components.

Fourier-transform infrared (FT-IR) spectroscopy can be used to identify the main peaks of a material's various functional groups and track their fluctuations within a fingerprinting area (Yeo et al. 2018). FT-IR was performed to investigate the interaction between CXB and various BLs components. The FT-IR spectra of PC, SDC, CXB, and the selected CXB-BLS formulation (CXB-BLs 7) are shown in Fig. 4. The corresponding spectra were collected at wavenumbers ranging from 4000 to 650 cm^−1^ (Abou Taleb et al. 2020).

FT-IR spectrum of PC showed a broad peak at 3312.27 cm^−1^, sharp peaks at 3009.78, 2922.52, and 2852.75 cm^−1^ (Ghanbarzadeh 2015), as well as several sharp peaks below 1800 cm^−1^ which can be attributed to the PO^−^ stretching vibrations (Arrondo et al. 1984). While FT-IR spectrum of SDC revealed three distinctive peaks at 2935.9, 2863.3, and 1562.2 cm^−1^, which can be contributed to CH stretching vibration and the COO − stretching vibration bands (Yang and Mantsch 1986; Yang et al. 2005). CXB FT-IR spectrum showed standard peaks at 1159 cm^−1^ which can be attributed to S = O stretching, 3338 cm^−1^ due to NH_2_ stretching and 1563 cm^−1^ contributed to N–H stretching (Gulshan et al. 2017). The FT-IR spectrum of free CXB revealed medium absorption bands at 3332 and 3225 cm^−1^, which correspond to the drug’s -NH symmetric and asymmetric stretching vibrations, respectively. The additional distinguishing bands can be attributed to the following group vibrations: 1157 and 1345 cm^−1^ (S = O symmetric and asymmetric stretching, respectively), 1562 cm^−1^ (NH bends), and 791 cm^−1^ (aromatic-CH bend) (Chawla et al. 2003; Pandya et al. 2009).

In contrast to the distinct peaks of free CXB, the optimized CXB-BLs formulation's FT-IR spectrum displayed a shift in intensity. Physical interactions between the medication and BLs components, such as dipole, hydrogen, or Van der Wall bonds, may be the cause of the shift in characteristic peaks. This leads to the best possible trapping of CXB in BLs without causing any chemical alterations to the drug’s structure following encapsulation (Darwish et al. 2024; Taleb et al. 2024).

## *In vitro* release study

**Fig.5:**
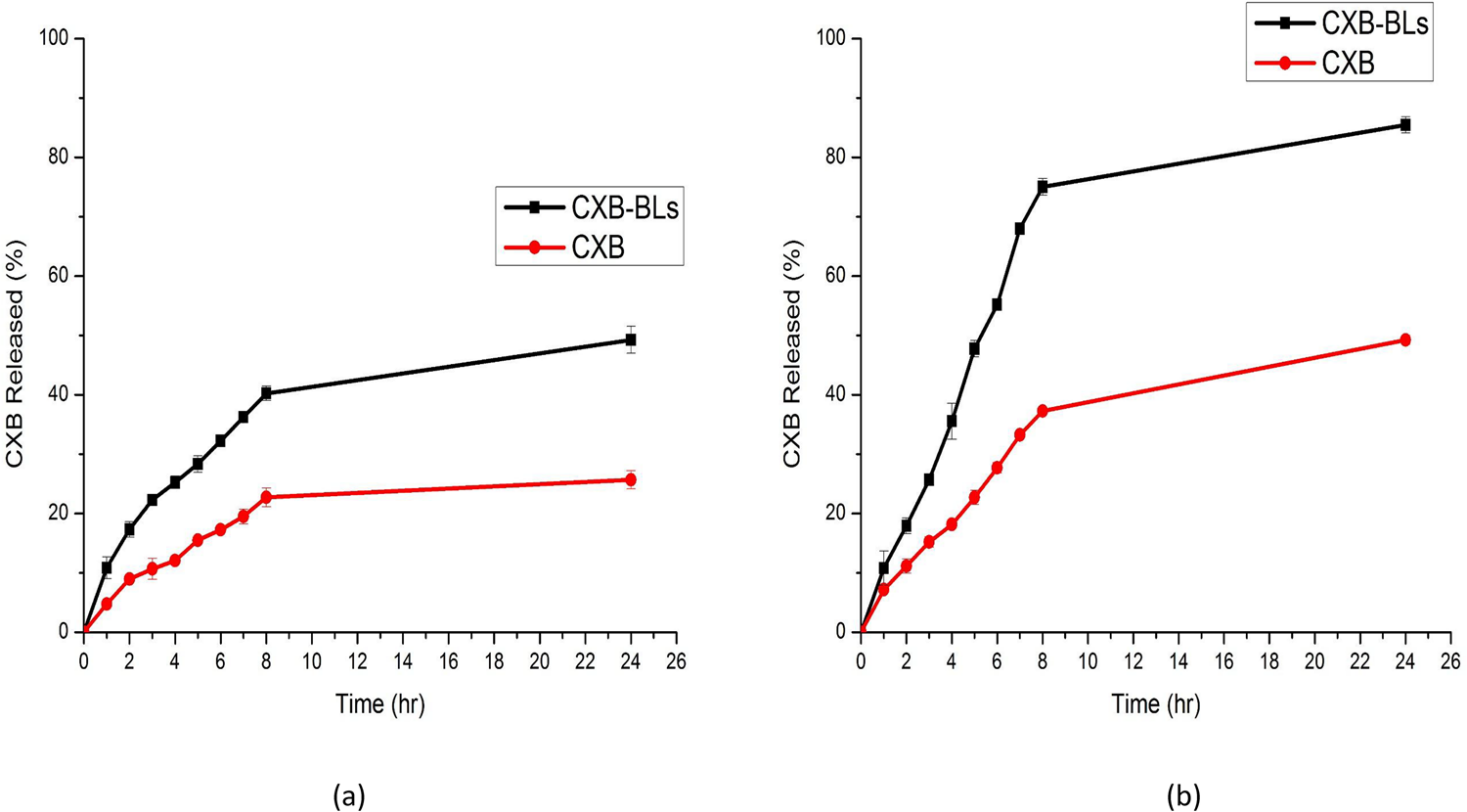
Release profiles of CXB from CXB-BLs formulation and free CXB suspension in (**a**)0.1 N HCl (pH 1.2) and (**b**) PBS (pH 6.8).

Figure 5 displayed the release profile of CXB from the selected CXB-BLs formulation and from the free CXB solution. The release study was performed in two release media: 0.1 N HCl (pH 1.2) and PBS (pH 6.8), to simulate the pH gradient of the gastrointestinal tract. Figure 5(a) showed the release profiles of free CXB solution and the selected CXB-BLs in 0.1 N HCl (pH 1.2). The results revealed that CXB release was slow from both free CXB solution and BLs dispersion, while BLs exhibited a faster release profile of 32.25.2 ± 0.87% after 6 h compared to 17.28 ± 0.88% in case of the plain CXB suspension. CXB is a weak acid molecule (Ahika et al. 2015) that has poor solubility and release in acidic medium. Incorporation of CXB in BLs enhanced its release in acidic medium as it has been previously mentioned that BL has the ability to increase drug solubility in the dispersion medium and vesicular lipid bilayer fluidization (Aburahma 2016).

Figure 5(b) showed the release profiles of free CXB solution and the selected CXB-BLs in PBS (pH6.8). The results revealed that release of CXB from free drug solution and BLs dispersion was faster in alkaline medium. BLs exhibited a biphasic release profile with a faster initial release of 55.2 ± 0.72% after 6 h compared to 27.72 ± 0.88% in case of the free CXB solution. Drug that had been adsorbed on the surface of CXB-BLs and incorporated among the fatty acid chains in the BLs bilayers may cause a rapid release of CXB from the BLs, resulting in the first burst release of drug from BLs in both release media (Abd-Elbary et al. 2008; Abd El-Alim et al. 2014). The initial burst release was followed by a steady, persistent release lasting up to 24 h. The increased drug release from the BLs dispersion may be owing to the generated small particles in the nanometric range with a significant surface area (Alshawwa et al. 2023). The feature of BLs as drug reservoirs that may release the encapsulated medicines at a continuous and controlled rate is responsible for the observed sustained release behavior of CXB from the developed BLs. (Chilkawar et al. 2015).

Table 3 displays the results of a linear regression analysis of the mathematical models used for CXB release data from the CXB-BLs in the two release mediums and from the Free CXB solution. In comparison to zero-order and first-order kinetic models, the results indicated that the correlation coefficient (*R*^2^) values of CXB and CXB-BLs exhibited greater fitting to Higuchi's model. The Peppas equation (Peppas and Sahlin 1989; Lokhande et al. 2013) was used to further investigate the CXB release mechanism. Good linearity was realized, and the n value showed that the diffusion followed anomalous (non-Fickian) diffusion (Peppas and Sahlin 1989).

**Table 3:** The calculated correlation coefficients and kinetics parameters of CXB release profile from the optimized BLs formulation and free drug solution

Code	Q_8h_ ± SD (%)	Zero order	First order	Higuchi	Peppas	
		R^2^			R^2^	N
CXB in HCL	22.74 ± 1.56	0.5505	0.5959	0.9176	0.9237	0.1982
CXB-BLs in HCL	40.25 ± 1.23	0.5958	0.6427	0.8642	0.9361	0.1478
CXB in PBS	37.29 ± 0.26	0.6286	0.6598	0.8907	0.9754	0.2452
CXB-BLs in PBS	75.04 ± 1.41	0.5603	0.6805	0.8939	0.9439	0.2082

*Q*_*8h*_ Percent total CXB released after 6 h

## *In vivo* Study

### Effect of CXB-BLs on behavioral activity and AChE

**Fig.6:**
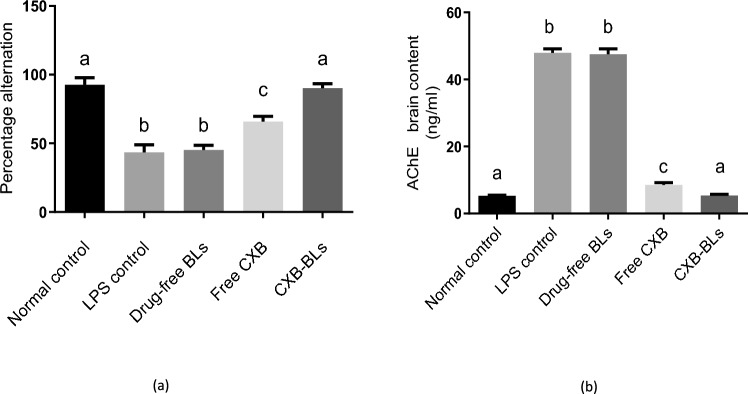
Effects of CXB-BLs on Behavioral Activity and AChE.Data were expressed as mean ±SD. Statistical analysis was carried out by one-way ANOVA followed by Tukey's multiple comparisons test. Smaller letter indicates non-significant difference whereas different letters indicate significant difference at p < 0.05.

Inflammation can be induced in a variety of ways, including the use of lipopolysaccharide (LPS). LPS is a molecule found in Gram-negative bacteria membrane which has an effect similar to AD as it can cause impaired spatial memory with an elevation in AChE release (Batista et al. 2019; Abbas et al. 2022). Figure 6a revealed the effect of different treatments on behavioral activity of mice. The results revealed that LPS reduced Y maze alteration by 53% when compared to negative control group. While treatment with free CXB and CXB-BLs significantly improved the behavioral activity by 51% and 108%, respectively, as compared with LPS group, in addition, treatment with CXB-BLs significantly improved the behavioral activity by 37%, as compared with Free CXB and returned it to its normal value.

Brain function depends on the balance of several neurotransmitter systems, including AchE (Watkins et al. 1994). The pathophysiology of learning and memory impairment in adult-onset dementia disorders, such as Alzheimer’s disease (AD), is associated with a deficient cholinergic neurotransmission function (Amenta et al. 2001). Figure 6b revealed that LPS administration increased AChE expression in brains by eightfold in comparison with negative control group. However, free CXB and CXB-BLs treatment decreased AChE in brains by 82%, and 89% respectively, as compared to LPS-treated mice. Furthermore, the treatment with CXB-BLs reduced AChE brain content by 37% as compared to free CXB treatment and returned its level to normal value. Previous studies mentioned that there is a strong relationship between AChE inhibition and improved cognitive function in AD patients (Wilkinson et al. 2004). Celecoxib has been demonstrated to inhibit the enzyme AChE in a non-competitive manner, probably due to its aromatic structural properties (Pohanka 2016). The results suggested that CXB-BLs exert neuroprotective effects, strengthen the memory and treat AD via its AChE inhibitory effect (Pohanka 2016). It has been previously mentioned that CXB ameliorated the cognitive decline in AD and it has a mitigating effect on learning and memory deficit (Guo et al. 2017). It has neuroprotective effect via its regulation of β-Amyloid and Heme Oxygenase-1 antioxidant (Mhillaj et al. 2020). Incorporation of CXB in BLs formulation increased its efficiency as BLs provide superior gastrointestinal stability compared to conventional therapies (Albash et al. 2019; Saifi et al. 2020). Furthermore, BS may function as intestinal permeability modifiers, resulting in an improved oral drug bioavailability following BLs encapsulation (Pavlović et al. 2018).

### Effect of CXB-BLs on TLR4 and IL-1β

**Fig.7:**
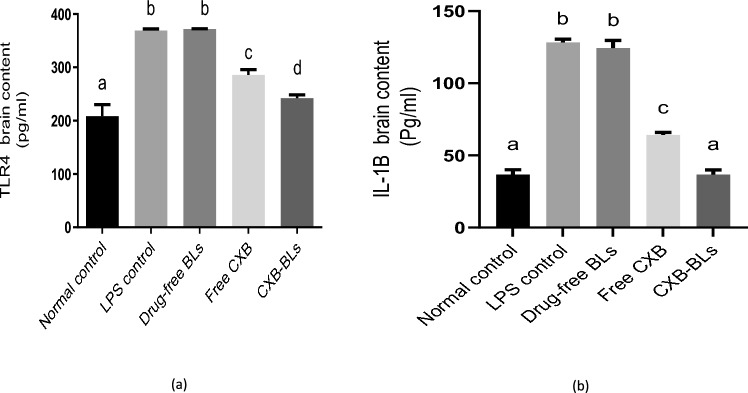
Effects of CXB-BLs on TLR4 and IL-1β.Data were expressed as mean ± SD. Statistical analysis was carried out by one-way ANOVA followed by Tukey's multiple comparisons test.Smaller letter indicates non-significant difference whereas different letters indicate significant difference at *p* < 0.05.

LPS induced inflammation via toll-like receptor 4 (TLR4) activation (Boonen et al. 2018). TLR4 activates myeloid differentiation transcription factors, stimulating a plethora of pro-inflammatory genes (Gray et al. 2011), with glial cells activation that express TLR4 (Chistyakov et al. 2018). Results illustrated in Fig. 7a revealed that LPS elevated TLR4 by 77% as compared to negative control group, while free CXB and CXB-BLs decreased it by 23% and 34% respectively as compared to LPS group. Moreover, the treatment with CXB-BLs decreased brain content of TLR4 by 15% as compared to free CXB treatment.

Neuroinflammation precedes neurodegenerative pathologies development such as AD (Wee Yong 2010). LPS is used in experimental *in vivo* models provoking neuro-inflammation and promoting amyloid deposition (Miklossy 2008). Results illustrated in Fig. 7b showed that LPS increased pro-inflammatory cytokine IL-1β by 2.5-fold as compared to negative control group. While free CXB and CXB-BLs decreased it by 50% and 71% respectively as compared to LPS group. These results suggested that CXB-BLs lowered microglial production of pro-inflammatory cytokines mediated by LPS decreasing gliosis via reducing the levels of IL-1β in the brain. The treatment with CXB-BLs reduced IL-1β brain content by 43% as compared to free CXB treatment and returned its level to normal value. These results confirmed that CXB-BLs formulation enhanced CXB efficiency in decreasing neuro-inflammation and neuro-degeneration in AD. It has been previously mentioned that CXB can mitigate Aβ-induced neurotoxicity through inhibiting neuro-inflammation and restoring the balance of apoptosis and neurogenesis which happen in AD (Jang and Surh 2005; Abdel-Aal et al. 2021).

#### Histopathological Examination

**Fig.8:**
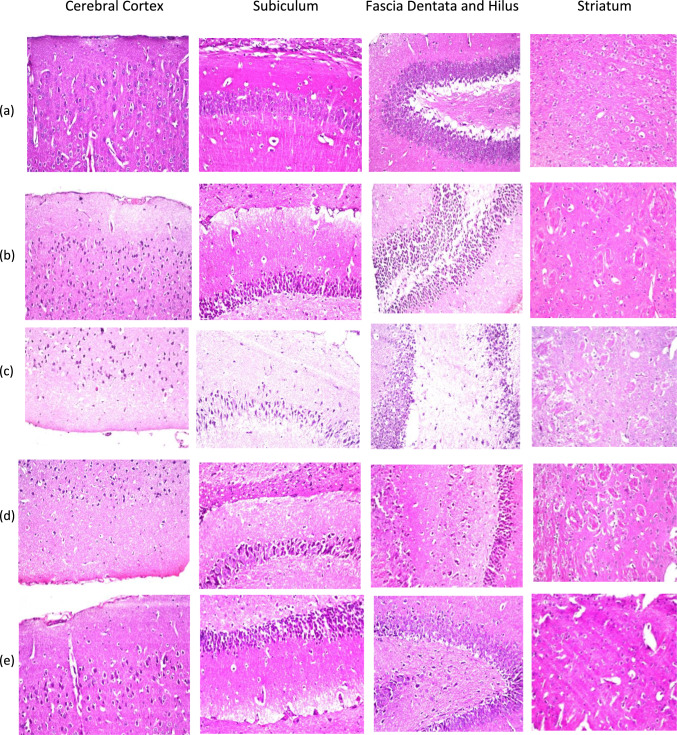
Photomicrographs of Cerebral Cortex, Subiculum, Hippocampus (Fascia Dentata and Hilus) and Striatum of **a** Negative control, **b** LPS group, **c** Drug-free BLs, **d** Free CXB and **e** CXB-BLs. All photomicrographs were taken at two magnification powers (×200 and ×400).

The histopathological examination of different tissues of brain of mice from different groups is illustrated in Fig. 8. The negative control group showed normal cerebral cortex, hippocampus (subiculum and fascia dentata and hilus), and striatum tissues with non-histopathological alterations (Fig. 8a). While histopathological examination of tissues from LPS group revealed the presence of nuclear pyknosis and degeneration in all of the neurons of the cerebral cortex, hippocampus (subiculum and fascia dentata and hilus). Along with neuronal degeneration and neuronophagia, the cerebral cortical blood vessels also showed focal lymphocytic infiltration with degenerated neurons, congestion with diffuse gliosis, and severe vasculitis. Figures also revealed degeneration in some of the neurons with multiple eosinophilic plagues formation in the striatum (Fig. 8b). Tissues of drug-free BLs group showed histopathological examination similar to that noticed with LPS group with nuclear pyknosis and degeneration in all of the neurons of the cerebral cortex, Hippocampus (subiculum and fascia dentata and hilus), degeneration in some of the neurons with multiple eosinophilic plagues formation in the striatum (Fig. 8c) which indicated that drug-free BLs has no anti-inflammatory activity.

It has been noticed that treatment with free CXB partially improved the tissues inflammations as there were nuclear pyknosis and degeneration in all of the neurons of the cerebral cortex, while there were nuclear pyknosis and degeneration in some neurons of the hippocampus (subiculum and fascia dentata and hilus). There were nuclear pyknosis and degeneration in some neurons with focal eosinophilic plagues formation in the striatum (Fig. 8d). Treatment with CXB-BLs showed that some neurons of the cerebral cortex showed nuclear pyknosis and degeneration, there were nuclear pyknosis and degeneration in few neurons of the subiculum while others returned to be normal. The figures also revealed normal fascia dentata and hilus and striatum tissues with non-histopathological alterations (Fig. 8e). Histopathological examinations supported the results of the inflammatory biomarkers as the anti-inflammatory efficiency of the developed BLs formulation improved the tissue examination.

Mouse models have been used to investigate the molecular pathways behind Alzheimer’s disease. To be deemed effective, such models must accurately reproduce the actual disease in as many aspects as possible, and mouse models of Alzheimer's disease have been partially successful in simulating the human situation. Some models have shown behavioral and neurophysiological abnormalities similar to those seen in humans, as well as neurophysiological problems, inflammation, and, on rare occasions, a decrease in the number of CA1 pyramidal neurons (Gotz et al. 2004). Furthermore, certain models exhibit a regional pattern of cerebral and vascular amyloid deposits (Götz and Ittner 2008) as well as the accumulation of Tau, P-tau, acetylated tau, reactive astrocytes, and microglia, which is comparable with the distribution of these events in real AD (Duyckaerts et al. 2008). Recent research suggests that multiple mouse models imitate the plaque pathology of Alzheimer’s disease and cause moderate behavioral impairments (Lee et al. 2005; Pádua et al. 2024). None, however, create the neurofibrillary tangles associated with Alzheimer’s disease or the severe behavioral changes that characterize the disease’s final stages (Li et al. 2011).

## Conclusion

Celecoxib-loaded bilosomes (CXB-BLs) were synthesized using the thin-film evaporation process. A 2^3^-factorial design was used to achieve an optimal CXB-BLs formulation. The produced CXB-BLs demonstrated high entrapment efficiency percentages (94.16 ± 1.91 to 98.38 ± 0.85%), vesicular size ranged from 241.8 ± 6.74 to 352 ± 2.34 nm, and negative zeta potential values. *In vitro* release profiles revealed that the improved BLs formulation released CXB at a higher and more sustained rate than the free medication for up to 24 h. *In vivo* study revealed that CXB-BLs treatment mitigates LPS-induced AD through improving the memory via its AChE inhibitory effect and inhibiting neuro-inflammation and neuro-degeneration through suppressing TLR4 and IL-1β. These findings showed that BLs have the potential to enhance CXB's anti-inflammatory activity for the oral treatment of Alzheimer’s disease.

## Data Availability

All data generated or analyzed during this study are included in this published article.
